# Quantum Multi-User Broadcast Protocol for the “Platform as a Service” Model

**DOI:** 10.3390/s19235257

**Published:** 2019-11-29

**Authors:** Peng Shi, Nachuan Li, Shumei Wang, Zhi Liu, Mengran Ren, Hongyang Ma

**Affiliations:** Quantum Physics Laboratory, School of Science, Qingdao University of Technology, Qingdao 266520, China; shipeng@qut.edu.cn (P.S.);

**Keywords:** Quantum Cloud Platform, phase-covariant cloning, Quantum Cloning Machine, multi-user broadcast, Platform as a Service

## Abstract

Quantum Cloud Computing is the technology which has the capability to shape the future of computing. In “Platform as a Service (PaaS)” type of cloud computing, the development environment is delivered as a service. In this paper, a multi-user broadcast protocol in network is developed with the mode of one master and *N* slaves together with a sequence of single photons. It can be applied to a multi-node network, in which a single photon sequence can be sent to all the slave nodes simultaneously. In broadcast communication networks, these single photons encode classical information directly through noisy quantum communication channels. The results show that this protocol can realize the secret key generation and sharing of multiple nodes. The protocol we propose is also proved to be unconditionally secure in theory, which indicates its feasibility in theoretical application.

## 1. Introduction

The interest in quantum cloud computing (see [1]) has really taken off in the past few years, but, in the future, quantum computers will be quite expensive in nature and will not be available to every one. To solve this problem, a basic idea of cloud computing, which migrates the processing power from customer’s computer to remote Internet servers, is put forward. One of the service models on quantum cloud computing, “Platform as a Service” [2] is proposed for supporting online development environment. Multi-user broadcast, similar to multicast, can be used on cloud platform to communicate between router and users.

Multicast has many applications such as access to business information dissemination, distributed databases, distance teleconferencing, and network learning. Multi-user broadcast protocol can increase quantum network efficiency and conserve its resources. A sender, Alice, wants to send some confidential information to receivers, Bob brothers. They must multicast communicating messages, on the basis of high enough confidentiality and legitimacy of information. As an applied system, its safety is very important.

A simple communication mode is one to one, such as the BB84 protocol [3]. Multiparty communication [4,5,6] has drawn much attention. Matsumoto [7] proposed a quantum-key-distribution protocol that could enable three parties to agree at once on a shared common random bit string in the presence of an eavesdropper without the use of entanglement, which might not be directly applied to the one-to-many multicast communication. Yan [8] proposed a quantum secret sharing protocol between multiparty *m* members in Group 1 and multiparty *n* members in Group 2 using a sequence of single photons. Another extension of the theory of various Quantum Cloning Machines (QCM) protocols [9,10,11,12,13,14] has been designed and their applications and implementations have been studied, both theoretically and experimentally. The research of quantum cloning and deep application are continuously developing. In our research, we use optimal one to M phase-covariant QCM to implement multi-user broadcast protocol. Theoretically, these QCM, which provide the most dangerous and efficient attack for the BB84 protocol, can be used to multicast message in an optimal fidelity.

The rest of this paper is organized as follows. We show a summary of relevant results concerning multicast addresses and quantum cloning in Section 2. In Section 3, we present multi-user broadcast protocol based on quantum cloning. In Section 4, we analyze optimal fidelity, throughput efficiency and security. Finally, we present our conclusions.

## 2. Overview

### 2.1. Multicast Addresses

**Figure 1:**
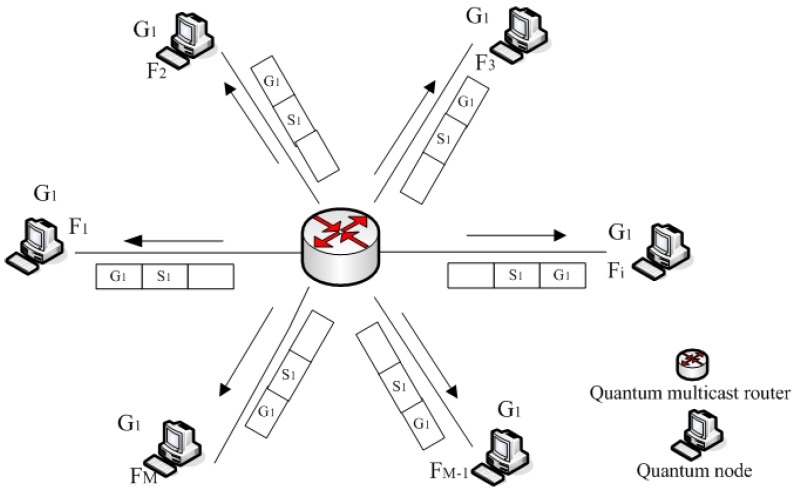
The simple multicast communication network.

There are one source and a group of destinations in multicast communication protocol. Figure 1 shows the simple multicast communication network, which depicts a set of quantum network nodes. The node of quantum network is a source sub of quantum data that must be delivered to a group $G_{1}$ of quantum network nodes, $F_{1}$, $F_{2}$, …, $F_{i}$, …, $F_{M}$, respectively. There is more than one quantum network node, but the group does not contain all possible quantum network nodes. This relationship is one to many.

The multicast address is a destination address for a group of quantum network nodes that have joined a multicast group, which is a great help to classical communication of quantum network communication protocol. A packet that uses a multicast address as a destination can reach all members of the group unless there are some filtering restrictions by the quantum network node. It only discusses the multicast addresses in the network layer, in particular the multicast addresses used in the IPv4 protocol. Multicast addresses for IPv6 can hardly even be touched. In TCP/IP protocol suites, Class D IP addresses are used as multicast addresses. The range of Class D addresses is 224.0.0.0–239.255.255.255, include 28 variable bits, $2^{28}$ (more than 268 million) multicast groups. Quantum network nodes may be permanent or transient. The former refers to the fact that the group has a permanently assigned address, rather than that members are permanently assigned to the group. The latter refers to the groups which do not have a permanent assignment to unreserved address.

### 2.2. Phase-Covariant Quantum Cloning

The no-cloning theorem [15] states that it is impossible to build a quantum copying machine that would perfectly copy arbitrary quantum states. However, we can try to clone a quantum state approximately with the optimal fidelity, or instead, we can try to clone it perfectly with the largest probability. Thus, various quantum cloning machines have been designed for different quantum information protocols. Some well-known quantum cloning machines include universal quantum cloning machine, phase-covariant cloning machine, the asymmetric quantum cloning machine, and the probabilistic quantum cloning machine. For instance, a cloning machine that achieves equal fidelity for every state is called a universal quantum cloning machine (UQCM). This problem is equivalent to distributing information to different receivers, and it is natural to require the performance is the same for every input state, since we do not have any specific information about the input state ahead. According to no-cloning theorem, it is expected that the original input state will be destroyed and become as one of the output copies. Because of the different types of copies, there are symmetric and asymmetric UQCMs. In the past years, much progress has been made in studying quantum cloning machines and their applications and implementations, both theoretically and experimentally. More details about quantum cloning are proposed in [16,17,18].

Here, we mainly discuss the phase-covariant quantum cloning, which has developed on the basis of universal quantum cloning and can produce equally good copies for all input states that lie on the equator of the Bloch sphere. The quantum state

(1) $$|\psi \rangle =\frac{1}{\sqrt{2}}\left(|0\rangle +e^{i\varphi }|1\rangle \right),$$

where φ∈[0,2π) is an arbitrary phase parameter, is often used as the input qubit of the phase-covariant QCM. For instance, the optimal 1→2 phase-covariant cloning transformation takes the form [19]

$${|0\rangle }_{A}{|0\rangle }_{B}{|0\rangle }_{C}\to \sqrt{\frac{1}{2}}\left({|0\rangle }_{B}{|00\rangle }_{AC}+{|1\rangle }_{B}{|\chi \rangle }_{AC}\right)={|\phi_{0}\rangle }_{ABC},$$

(2) $${|1\rangle }_{A}{|0\rangle }_{B}{|0\rangle }_{C}\to \sqrt{\frac{1}{2}}\left({|1\rangle }_{B}{|11\rangle }_{AC}+{|0\rangle }_{B}{|\chi \rangle }_{AC}\right)={|\phi_{1}\rangle }_{ABC},$$

where $|\chi \rangle =1/\sqrt{2}\left(|10\rangle +|01\rangle \right)$, A is the initial state of the cloning machine, B is an ancilla state of the system, and C is the blank state. The optimal fidelity of phase-covariant QCM is $F=1/2+1/\sqrt{8}\approx 0.85$, which is higher than the fidelity (F≈0.83) of UQCM.

Then, considering the optimal 1→M phase-covariant QCM, one of the cloning transformations is

(3) $$|\psi \rangle ⊗\left({|R\rangle }^{⊗M-1}\right)⊗|M\rangle \overset{U_{1,M}}{\to }{|\psi \rangle }^{⊗M}⊗|M\left(\psi \right)\rangle ,$$

where |ψ〉 is the state of Hilbert space *H*, |R〉 is a blank state, and |M〉 is the state of auxiliary system (ancilla). The $U_{1,M}$ is described by the following unitary operator [20]:

(4) $$\begin{array}{cc} {|\phi_{0}\rangle }_{AC} & =U_{1,M}|0\rangle ⊗|R\rangle =\sum_{j=0}^{M-1}\alpha_{j}\left|\left(M-j\right)0,j1\rangle ⊗\right|R_{j}\rangle ,\\ {|\phi_{1}\rangle }_{AC} & =U_{1,M}|1\rangle ⊗|R\rangle =\sum_{j=0}^{M-1}\alpha_{M-1-j}\left|\left(M-1-j\right)0,\left(j+1\right)1\rangle ⊗\right|R_{j}\rangle , \end{array}$$

in which $\alpha_{j}=\sqrt{2(M-j)/M(M+1)}$, $|R\rangle$ is the initial state of the copy machine and the M−1 blank copies, and $|R_{j}\rangle \equiv |(M-1-j)0,1j\rangle$ are orthogonal normalized internal states of the QCM. With the help of an ancilla qubit, the optimal fidelity of 1→M phase-covariant QCM for equatorial qubits takes the form [18]

(5) $$F_{1,M}=\left\{\begin{array}{cc} 1/2+\sqrt{M(M+2)}/4M, & M\;\mathrm{is}\;\mathrm{even},\\ 1/2+(M+1)/4M, & M\;\mathrm{is}\;\mathrm{odd}, \end{array}\right.$$

which is the decreasing function for *M* and is better than the fidelity of UQCM via numerical computation.

## 3. Multi-User Broadcast Protocol Based on QCM

### 3.1. Neighbor Quantum Node Discovery Process

The quantum multicast router starts to discover its neighbors with probe packet messages, which contain the important informations: a list of addresses for neighbors from which the originating router has received probe packet messages, a generation ID used to detect changes in status of neighbors, and so on. After receiving the probe packet messages, the quantum multicast router records the address of the original router and the interface of received messages.

### 3.2. Neighbor Quantum Node Pruning Process

**Figure 2:**
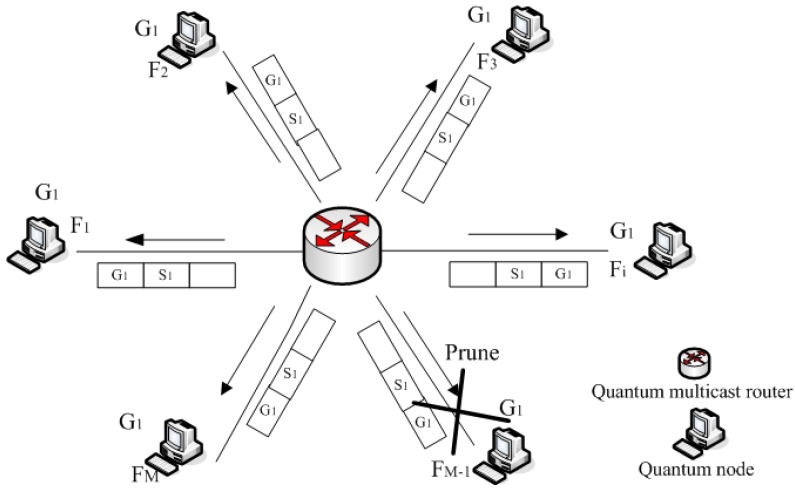
Pruning process of an undependable neighbor quantum node.

Probe packet messages are also used as keepalives when a neighbor has been discovered, and then the neighbor node send messages to the quantum multicast router within strict time intervals. If the quantum multicast router does not receive messages from this node after several probes within a specified period of time, the neighbor node will be declared dead. We call this step a neighbor quantum node pruning process, and this node is considered undependable at this time. For example, the pruning process of neighbor node $F_{M-1}$ is shown in Figure 2. The quantum multicast router must store the states of all nodes after each pruning process. If necessary, nodes and the router need to repeatedly send and receive messages in this process as many times as possible to complete the pruning process of quantum nodes.

### 3.3. Group Routing Tables Building Process

The quantum multicast router needs to collect members’ information and share it with other multicast routers, and then construct group routing table containing group members’ information. The graph of quantum nodes and links is called tree, so the quantum multicast router can be regarded as the root node. All other nodes $F_{i}\left(i=1,2,\cdots ,M\right)$ can only be reached from the root node through a single path. Multicast communication means that a sender sends messages to a group of recipients who are members of the same group. Since a copy of the message is sent by the sender and then copied and forwarded by the router, each multicast router needs to know the list of groups.

The group routing tables carry four core data: the quantum nodes identity, the list of links, a sequence number, and the age. The quantum nodes identity and the list of links are needed to make the quantum topology. The sequence number distinguishes new routing tables from old ones. The age prevents old routing tables from remaining in the domain for a long time. When the topology of a domain changes, any quantum nodes in the domain are quickly notified by the router to update their topology.

### 3.4. Multicast Date Packet Communication on Quantum Cloning Process

**Figure 3:**
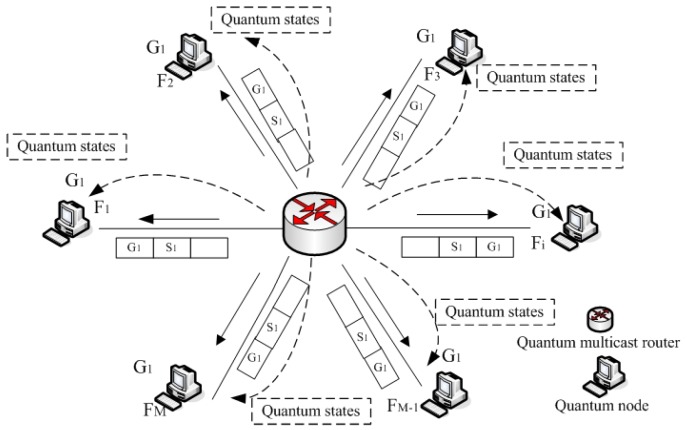
Multicast quantum cloning process.

We apply the phase-covariant cloning machine to multicast communication (in Figure 3). For each input single photons, we use a unitary transformation matrix *A* to prepare the input state on the equator of the Bloch sphere:

(6) $$A=\frac{1}{\sqrt{2}}\left(\begin{array}{cc} 1 & e^{-i\varphi }\\ e^{i\varphi } & -1 \end{array}\right).$$

The quantum multicast router encodes these strings as a block of (4n+δ), and sends $|\Psi_{0}\rangle =\sum_{i=0}^{4n+\delta }|\psi_{i}\rangle$ to $F_{i}\left(i=1,2,\cdots ,M\right)$ by quantum cloning via the quantum communication channel. For simplicity, we assume that the quantum channel is noiseless. The quantum multicast router transmits $|\Psi_{0}\rangle$ through quantum channel to construct QCM.

The quantum multicast router acts on an input state $|\psi \rangle$ as follows:

(7) $$\begin{array}{cc} \sum_{i=0}^{4n+\delta }U_{1,M}|\psi_{i}\rangle ⊗|R\rangle & =\sum_{i=0}^{4n+\delta }\sum_{j=0}^{M-1}\alpha_{M-1-j}|\left(M-1-j\right)\psi_{i},\left(j+1\right)\psi_{i}^{⊥}\rangle ⊗|R_{j}\left(\psi_{i}\right)\rangle \\ & =\sum_{i=0}^{4n+\delta }\sum_{j=0}^{M-1}\alpha_{M-1-j}|\left(M-1-j\right)\psi_{i},\left(j+1\right)\psi_{i}^{⊥}\rangle ⊗|\left(M-1-j\right)\psi_{i}^{*},\left(j+1\right){\left(\psi_{i}^{*}\right)}^{⊥}\rangle , \end{array}$$

where $|R_{j}\left(\psi_{i}\right)\rangle$ represents the internal state of QCM with $|R_{j}\left(\psi_{i}\right)\rangle ⊥|R_{k}\left(\psi_{i}\right)\rangle$ for all j≠k, and the equatorial qubits take the forms

(8) $$\begin{array}{cc} |\psi_{i}\rangle & =\frac{1}{\sqrt{2}}\left(|0\rangle +e^{i\varphi }|1\rangle \right),\\ |\psi_{i}^{*}\rangle & =\frac{1}{\sqrt{2}}\left(|0\rangle +e^{-i\varphi }|1\rangle \right),\\ |\psi_{i}^{⊥}\rangle & =\frac{1}{\sqrt{2}}\left(e^{-i\varphi }|0\rangle -|1\rangle \right),\\ |{\left(\psi_{i}^{*}\right)}^{⊥}\rangle & =\frac{1}{\sqrt{2}}\left(e^{i\varphi }|0\rangle -|1\rangle \right). \end{array}$$

Quantum correlation is the key concept for the quantum computation, quantum processing, and quantum information. Entanglement is a special case of quantum correlation. It is a property of correlations between two or more quantum systems. This nonlocal nature of entanglement has also been identified as an essential resource for many novel tasks. The preparation of entangled states in different physical systems has been widely studied and constitutes an essential step in many quantum information processing and transmission tasks [21,22,23]. For Alice and *M* Bob brothers, we assume that they all share a multi-particle entangled state $|\Omega \rangle$, and a choice of $|\Omega \rangle$ with these properties in the following *M*-qubit state:

(9) $$|\Omega \rangle =\frac{1}{\sqrt{2}}\left(|0\rangle ⊗{|\phi_{0}\rangle }_{AC}+|1\rangle ⊗{|\phi_{1}\rangle }_{AC}\right),$$

where ${|\phi_{0}\rangle }_{AC}$ and ${|\phi_{1}\rangle }_{AC}$ are the optimal cloning states given by Equation (4), and $|\Omega \rangle$ turns into

(10) $$|\Omega \rangle =\frac{1}{\sqrt{2}}(|0\rangle \sum_{j=0}^{M-1}\alpha_{j}|\left(M-j\right)0,j1\rangle ⊗|\left(M-1-j\right)0,j1\rangle +|1\rangle \sum_{j=0}^{M-1}\alpha_{{}_{j}}|j0,\left(M-j\right)1\rangle ⊗|j0,\left(M-1-j\right)1\rangle ).$$

The tenser product of $|\Omega \rangle$ with the equator qubits $|\Psi_{0}\rangle$ held by the (2M+4n+δ+1)-qubit state of Alice. Alice performs a joint measurement of the system $|\Psi_{0}\rangle ⊗|\Omega \rangle$ by using Bell measurement [24], where the four Bell states are defined as usual as the following

(11) $$\begin{array}{c} |\phi_{\pm }\rangle =\frac{1}{\sqrt{2}}\left(|00\rangle \pm |11\rangle \right),\\ |\psi_{\pm }\rangle =\frac{1}{\sqrt{2}}\left(|01\rangle \pm |10\rangle \right). \end{array}$$

Once one of the Bell states is obtained, we can recover the correct state by exploiting the symmetries of states ${|\phi_{0}\rangle }_{AC}$ and ${|\phi_{1}\rangle }_{AC}$ under the unitary transformation. Consider three Pauli matrices; the unitary transformation can be expressed as

(12) $$\sigma_{0}=\left(\begin{array}{cc} 1 & 0\\ 0 & 1 \end{array}\right),\sigma_{x}=\left(\begin{array}{cc} 0 & 1\\ 1 & 0 \end{array}\right),\sigma_{z}=\left(\begin{array}{cc} 1 & 0\\ 0 & -1 \end{array}\right).$$

The quantum multicast router measures Bell state of *A*, and the router and each nodes can transmit qubit BC in the following four forms as $\sum_{i=0}^{4n+\delta }\left(|0\rangle \pm e^{i\varphi }|1\rangle \right)/\sqrt{2}$ or $\sum_{i=0}^{4n+\delta }\left(|1\rangle \pm e^{i\varphi }|0\rangle \right)/\sqrt{2}$. When $\sum_{i=0}^{4n+\delta }\left(|1\rangle \pm e^{i\varphi }|0\rangle \right)/\sqrt{2}$ is obtained, the unitary transformation is $\sigma_{x}\left(\sigma_{x}\sigma_{z}\right)$ corresponding to the symbol “+”(“–”) in the equations. When $\sum_{i=0}^{4n+\delta }\left(|0\rangle \pm e^{i\varphi }|1\rangle \right)/\sqrt{2}$ is obtained, the unitary transformation is $\sigma_{0}\left(\sigma_{z}\right)$ corresponding to the symbol “+”(“–”). After these operations, the secret key is transmitted to the server, and the quantum multicast router and each node releases part of the quantum information. If the test is correct, the communication station (STA) is a legitimate user. Otherwise, there must be illegal eavesdroppers, which we discuss in the next section. In this process, quantum multicast router transmits classical information through Ethernet addresses. The main problem below is to change the three right-most bytes of the multicast IP address to hexadecimal. If the left-most number is greater than or equal to 8, subtract 8 from the left-most number. After the system gets the result, add the result to the starting Ethernet multicast address. Thus, the multicast date packet communication on quantum cloning process has completed successfully.

### 3.5. Selective Repeating Process

In this protocol model, the multicast router node acts as a key management system and authenticates the communication users in communication. The multicast router manages the security key for the communication users and authenticates the identity of users by arbitrating the quantum signature using the shared quantum state [25]. In the process of communication, the multicast router node conducts authentication occasionally to prevent the user from being attacked.

Ideally, we assume that each photon emission is perfect. If the information transmission fails due to channel loss or eavesdropping, the multicast router will return a negative acknowledgment (NAK) to the nodes. After receiving the NAK, the node will start the selective repeating process, thus ensuring the security and reliability of the communication. The strings are transmitted continuously as a block of (4n+δ), the quantum multicast router resends (or repeats) only those codewords that are negatively acknowledged. Since the strings must be delivered to the user in correct order, a buffer must be provided at the receiver to store the error-free qubits of received qubits after error detection. When the first negatively acknowledged strings are successfully received, the receiver releases the error-free qubits in consecutive order until the next erroneously received qubits are encountered. Sufficient qubits receiver buffers must be provided, otherwise the qubits buffers may overflow and quantum data may be lost.

## 4. Analysis

### 4.1. Analysis of Quantum Bit Error Rate and Secure Key Rate

The Quantum Bit Error Rate (QBER) is defined as the number of wrong bits to the total number of received bits and is normally in the order of a few percent. In the following, we use it expressed as a function of rates:

(13) $$QBER\approx \frac{R_{error}}{R_{sift}},$$

where the sifted key corresponds to the cases in which Alice and Bob made compatible choices of bases, hence its rate is half that of the raw key. In a practical quantum key distribution system, e.g. the BB84 protocol, after attenuation and sifting, the sifted key generation rate is given by [26]

(14) $$R_{sift}=\frac{1}{2}q\cdot f_{rep}\cdot \mu \cdot t_{link}\cdot \eta .$$

where the factor *q* (q≤1, typically 1 or $\frac{1}{2}$ ) must be introduced for some phase-coding setups in order to correct for noninterfering path combinations, $f_{rep}$ is the pulse rate, μ is the mean number of photon per pulse, $t_{link}$ is the probability of a photon to arrive at the analyzer, and η is the probability of the photon being detected.

The secure key rate in our protocol is the quantum communication rate of the whole system. This depends on the rate of key distribution when each root node communicates with its children, that is the rate of the sifted key generation.

### 4.2. Analysis of Optimal Fidelity

The fidelity is widely used within the quantum computation and quantum information community, and we discuss the quantum multi-user broadcast protocol for the “Platform as a Service” model. In our study, the algorithm discussed above is sufficient to complete each step of the computation with higher fidelity than 1→M phase-covariant QCM by Equation (5).

The quantum multicast router obtains measurement outcome for ${|\Psi_{0}\rangle }_{out}$, and publicly announces the results. $S_{1}$, $S_{2}$, …, $S_{i}$, …, $S_{N}\left(N<M\right)$ carry out the unitary transformation σ separately on their qubits. The final states of qubits for $S_{i}\left(i=1,2,\cdots ,N\right)$ equal to the original state $|\Psi_{0}\rangle =\frac{1}{\sqrt{2}}\left(|0\rangle +e^{i\varphi }|1\rangle \right)$. We now wish that the optimal phase-covariant cloning machine can be achieved. Let us see fidelity, which is found to take the form

(15) $$F=\frac{1}{2}\left[1+\eta \left(1,N\right)\right]=\left\{\begin{array}{cc} 1/2+\sqrt{N(N+2)}/4N, & N\;\mathrm{is}\;\mathrm{even},\\ 1/2+(N+1)/4N, & N\;\mathrm{is}\;\mathrm{odd}, \end{array}\right.$$

where

(16) $$\eta \left(1,N\right)=\sum_{j=0}^{N-1}\alpha_{j}\alpha_{N-1-j}\frac{C_{N-1}^{j}}{\sqrt{C_{N}^{j}C_{N}^{j+1}}}.$$

### 4.3. Analysis of Throughput Efficiency

Next, we discuss the throughput efficiency which is defined as the ratio of the average number of information digits successfully accepted by the receiver per unit of time to the total number of digits that could be transmitted per unit of time. Suppose the simple case of the communication protocol, and the sender continuously sends codewords to the receiver and only resends those negatively acknowledged codewords. First, three probabilities are defined: $P_{c},P_{d}$, and $P_{e}$, where $P_{c}+P_{d}+P_{e}=1$. $P_{c}$ is the probability of receiving no error message, $P_{d}$ is the probability of receiving detectable error pattern, and $P_{e}$ is the probability of receiving undetectable error pattern. Then, the probability of receiving vector being accepted by the receiver is $P=P_{c}+P_{e}$.

The average number of retransmissions (including original transmissions) for a codeword to be successfully received by the receiver is

(17) $$t_{AV}=1\cdot P+2\cdot P\cdot \left(1-P\right)+\cdots +l\cdot P\cdot {\left(1-P\right)}^{l-1}+\cdots =\frac{1}{P}$$

Finally, the throughput of sending n codewords successfully is $T=\frac{n}{t_{AV}}=nP$. Thus, the throughput efficiency depends on the channel error rate only.

### 4.4. Analysis of Security under Typical Attack

The Security of this broadcast protocol depends on every process of communication between one source and a group of destinations. The protocol is divided into five parts as neighbor quantum node discovery process, neighbor quantum node pruning process, group routing tables building process, multicast date packet communication on quantum cloning process, and selective repeating process. Conventional data communication is used in processing classical information, and cleartext can be transferred through quantum channel. There is no information revealed. As for the vector attack, according to quantum no-cloning theorem, the attacker cannot accurately copy quantum nodes for DOS attack. If the attacker generates illegal users to prevent information transmission, it will be found in the authentication process by the key management system and the illegal communication will be terminated.

#### 4.4.1. Attack via Direct Measurement

One can analyze the security of multicast data packet communication via direct measurement. $F_{i}\left(i=1,2,\cdots ,M\right)$ receives the random (4n+δ) qubits, who measures each qubits in the basis $\sigma_{x}$ or $\sigma_{z}$ at random. $S_{i}\left(i=1,2,\cdots ,N\right)$ receives $\varepsilon (|\Psi_{0}\rangle \langle \Psi_{0}|)$, where ε describes the quantum operation due to the combined effect of the channel and eavesdropper’s (Eve) actions. $S_{i}\left(i=1,2,\cdots ,N\right)$ then publicly announces this fact. For now, each of the N+1 nodes has its own states described by separate density matrices. Note that, at this point, since $S_{0}$ did not reveal *b*, Eve has no knowledge of what basis she should have measured to eavesdrop in the communication. At best, she can only guess. If her guess were wrong, then she would have disturbed the state received by $S_{i}\left(i=1,2,\cdots ,N\right)$. Moreover, whereas in reality the noise ε may be partially due to the environment in addition to Eve’s eavesdropping, it does not help Eve to have complete control over the channel. Thus, Eve is entirely responsible for ε.

When the quantum multicast router first receives a multicast packet from $S_{1}$, the packet check is performed, using the routing table to verify that the packet arrived on the right interface for the packet’s source. If the packet arrived on any other interface, drop it.

#### 4.4.2. Attack via Ancilla Particle

We suppose Eve intercepts the particle sent by Alice, which will be entangled with an ancilla $|e\rangle$ prepared by Eve. The unitary transformation that is implemented on Alice’s particle *E* does not change the state of single photons [27].

(18) $$\begin{array}{cc} E⊗|0e\rangle & =a|0e_{00}\rangle +b|1e_{0}1\rangle ,\\ E⊗|1e\rangle & =b^{\prime }|0e_{00}\rangle +a^{\prime }|1e_{0}1\rangle ,\\ E⊗|+e\rangle & =\frac{1}{2}[|+\rangle \left(a|e_{00}\rangle +b|e_{01}\rangle +b^{\prime }|e_{10}\rangle +a^{\prime }|e_{11}\rangle \right)+|-\rangle \left(a|e_{00}\rangle -b|e_{01}\rangle +b^{\prime }|e_{10}\rangle -a^{\prime }|e_{11}\rangle \right],\\ E⊗|-e\rangle & =\frac{1}{2}[|+\rangle \left(a|e_{00}\rangle +b|e_{01}\rangle -b^{\prime }|e_{10}\rangle -a^{\prime }|e_{11}\rangle \right)+|-\rangle \left(a|e_{00}\rangle -b|e_{01}\rangle -b^{\prime }|e_{10}\rangle +a^{\prime }|e_{11}\rangle \right], \end{array}$$

where the unitary transformation *E* can be written as

(19) $$E=\left(\begin{array}{cc} a & b^{\prime }\\ b & a^{\prime } \end{array}\right),$$

where ${\left|a\right|}^{2}={\left|a^{\prime }\right|}^{2}$, ${\left|b\right|}^{2}={\left|b^{\prime }\right|}^{2}$, and ${\left|a\right|}^{2}+{\left|b\right|}^{2}=1$. Thus, the probability of Eve being detected is

(20) $$P_{e}={\left|b\right|}^{2}=1-{\left|a\right|}^{2}={\left|b^{\prime }\right|}^{2}=1-{\left|a^{\prime }\right|}^{2},$$

the eavesdropping brings a certain amount of error rate, and it must be detected.

According to the information theory, the amount of maximum accessible information in quantum system is limited by Holevo limit:

(21) $$\chi \left(\psi \right)=S\left(\psi \right)-\sum_{i}p_{i}S\left(\psi_{i}\right),$$

where S(ψ) is the von Neumann entropy of state ψ, $\psi =\sum_{i}p_{i}\psi_{i}$, and $\psi_{i}$ is a state prepared in probability $p_{i}$. If communicating parties prepare states $|0\rangle$, $|1\rangle$, $|+\rangle$ and $|-\rangle$, then the information entropy $H\left(p\right)=-\sum_{i}p_{i}log_{2}p_{i}=2$. Thus the von Neumann entropy of Eve [27] is $S\left(\psi^{\prime }\right)=0<H\left(P\right)$. It seems that Eve cannot obtain the complete information of photons in our protocol.

## 5. Conclusions

We describe a multi-user broadcast protocol in network for the one-to-many multicast communication network including the master and *N* slave mode using a sequence of single photons. This protocol might be useful in practice because it guarantees multicast information robustness. In the one-to-many multicast communication mode, $S_{0}$ creates (4n+δ) random bits and multicasts information to $S_{i}\left(i=1,2,\cdots ,N\right)$. $S_{0}$ and $S_{i}$ publicly announce the selection of the random measurement basis. There are at least 2*n* bits left, and if not, the protocol will be aborted. Meanwhile, the Calderbank–Shor–Steane (CSS) coding theory can be employed for correcting the errors introduced by the noisy communication channel. Therefore, the N+1 nodes compute the related information, and finally obtains the correct key. The commercial success of quantum key distribution for the generation of a private shared secret key motivates this investigation. The protocol is also proved to be unconditionally secure in theory, which indicates its feasibility in theoretical application. For future study, it may be significant to investigate the performance of our protocol for encoding secret classical messages.

In our proposed protocol, the photon is the carrier of information. Quantum information is encoded in the flying photon bit, and the transmit power is related to the performance of the transmitter module. In practical quantum communication, the transmission distance is limited due to the imperfection of the transmitter module and the detection module, which is a general problem in all practical quantum communication systems.

This protocol mainly establishes the quantum multi-user communication model without considering channel noise. In the practical noisy channel, we can use the quantum error-correction code to correct the error generated in the transmission process. Commonly used quantum error-correction codes are Quantum Stabilizer Code (QSC) and Quantum Low-Density Party-Check Code (QLDPC) [28].

One thing to point out is that we concentrate only on closed systems where the decoherence and dissipations are neglected. It is well-known that in open quantum systems the Hamilton operator is non-Hermitian [29,30]. The dynamical behavior of open quantum systems plays a key role in many applications of quantum mechanics, such as the environment-induced decay of quantum coherence, relaxation in many-body systems, and applications in condensed matter theory, quantum transport, quantum chemistry, and quantum information. If the decoherence and dissipation of the open systems are considered, the protocol based on quantum error correction coding needs to be studied in the future in more detail.
